# Results of the Cologne Corona Surveillance (CoCoS) project – a cross-sectional study: Survey data on nonadherence to recommended individual behaviours for SARS-CoV-2 pandemic containment

**DOI:** 10.3205/000353

**Published:** 2025-11-24

**Authors:** Max Oberste, Kilian Baumann, Kija Shah-Hosseini, Michael Buess, Kerstin Daniela Rosenberger, Annelene Kossow, Felix Dewald, Jomana Reusch, Teodora Asenova, Florian Neuhann, Martin Hellmich

**Affiliations:** 1 Institute of Medical Statistics and Computational Biology, Medical Faculty and University Hospital of Cologne, University of Cologne, Germany; 2 Cologne Health Authority, Cologne, Germany; 3 Institute of Hygiene, University Hospital of Muenster, University Muenster, Germany; 4 Institute of Virology, Medical Faculty and University Hospital of Cologne, University of Cologne, Germany; 5 Heidelberg Institute of Global Health, University Heidelberg, Germany; 6 School of Medicine and Clinical Sciences, Levy Mwanawasa Medical University, Lusaka, Zambia

**Keywords:** SARS-CoV-2, vaccination, countermeasures, hygiene, contact tracing, surveys and questionnaires

## Abstract

**Background:**

To curb SARS-CoV-2 transmission, citizens were urged to get vaccinated and adhere to hygiene recommendations (keeping distance, washing hands, wearing face masks), as well as informing contacts if infected. Studies confirm the effectiveness of these measures in reducing virus spread. Understanding factors influencing nonadherence is vital for enhancing the efficacy of future pandemic education campaigns.

**Methods:**

An online survey assessed nonadherence to SARS-CoV-2 containment recommendations among 30,000 randomly selected Cologne residents aged 18 or older. Invitations were sent on December 7, 2022, with the survey open until January 7, 2023. Logistic regression analyzed associations between participants’ sociodemographic, health, and virus-related characteristics and reported nonadherence to recommended behaviors.

**Results:**

Out of 30,000 invited Cologne residents, 4,486 (15%) responded, with 10% reporting not having received recommended SARS-CoV-2 vaccination. Nonadherence correlated significantly with older age, male gender, low income, living alone, migrant background, and chronic lung disease. 80% reported not adhering to current hygiene recommendations, linked to younger age, absence of certain pre-existing conditions, and prior SARS-CoV-2 infection. Additionally, 16% reported hesitancy in informing all contacts upon testing positive, associated with male gender, absence of certain pre-existing conditions, and fewer booster doses.

**Conclusion:**

The results presented here point to which sociodemographic, health, and virus-related factors are associated with nonadherence to recommended individual behaviors to mitigate the SARS-CoV-2 pandemic. However, issues related to the representativeness of the sample for the general population limit the validity of the results.

Trial registration: DRKS.de, German Clinical Trials Register (DRKS), identifier: DRKS00024046, registered on 25 February 2021

## Zusammenfassung

**Hintergrund:**

Um die Übertragung von SARS-CoV-2 einzudämmen, wurden die Bürger aufgefordert, sich impfen zu lassen und die Hygieneempfehlungen zu befolgen (Abstand halten, Hände waschen, Alltagsmasken tragen) sowie Kontaktpersonen zu informieren, wenn sie infiziert sind. Studien bestätigen die Wirksamkeit dieser Maßnahmen zur Verringerung der Virusausbreitung. Um die Wirksamkeit künftiger Pandemieaufklärungskampagnen zu verbessern, ist es wichtig, die Faktoren zu verstehen, die die Nichteinhaltung beeinflussen.

**Methodik:**

Eine Online-Umfrage untersuchte die Nichteinhaltung der Empfehlungen zur Eindämmung von SARS-CoV-2 unter 30.000 zufällig ausgewählten Kölner Bürgerinnen und Bürgern ab 18 Jahren. Die Einladungen wurden am 7. Dezember 2022 verschickt, und die Umfrage war bis zum 7. Januar 2023 geöffnet. Mittels logistischer Regression wurde der Zusammenhang zwischen den soziodemografischen, gesundheitlichen und virusbezogenen Merkmalen der Teilnehmerinnen und Teilnehmern und der angegebenen Nichteinhaltung der empfohlenen Verhaltensweisen analysiert.

**Ergebnisse:**

Von 30.000 eingeladenen Kölnerinnen und Kölnern antworteten 4.486 (15%), wobei 10% angaben, die empfohlene SARS-CoV-2-Impfung nicht erhalten zu haben. Die Nichtbefolgung korrelierte signifikant mit höherem Alter, männlichem Geschlecht, niedrigem Einkommen, Alleinleben, Migrationshintergrund und chronischer Lungenerkrankung. 80% gaben an, sich nicht an die aktuellen Hygieneempfehlungen zu halten, was mit jüngerem Alter, dem Fehlen bestimmter Vorerkrankungen und einer früheren SARS-CoV-2-Infektion zusammenhing. Darüber hinaus gaben 16% an, dass sie zögerten, alle Kontaktpersonen nach einem positiven Test zu informieren, was mit dem männlichen Geschlecht, dem Fehlen bestimmter Vorerkrankungen und weniger Auffrischungsimpfungen zusammenhing.

**Schlussfolgerung:**

Die hier vorgestellten Ergebnisse deuten darauf hin, welche soziodemografischen, gesundheitlichen und virusbezogenen Faktoren mit der Nichteinhaltung der empfohlenen individuellen Verhaltensweisen zur Eindämmung der SARS-CoV-2-Pandemie in Zusammenhang stehen. Die Aussagekraft der Ergebnisse wird jedoch durch Probleme im Zusammenhang mit der Repräsentativität der Stichprobe für die Allgemeinbevölkerung eingeschränkt.

**Registrierung der Studie:**

DRKS.de, Deutsches Register Klinischer Studien (DRKS), Identifikator: DRKS00024046, registriert am 25. Februar 2021

## Introduction

Since outbreak in 2019, coronavirus disease 2019 (COVID-19) has had a significant global impact on societies, economies, and public health. As of March 2024, approximately 38.4 million infections and 175 thousand deaths have been recorded in Germany alone [1]. These figures highlight the challenge posed by the virus and the importance of identifying and implementing effective infection control measures.

To reduce the spread of the virus, people in Germany were asked to adapt their personal behaviour according to expert recommendations. They were invited to take free SARS-CoV vaccinations [2] and advised to follow standard hygiene recommendations and, if possible, to inform all contacts if they were infected with SARS-CoV-2 [3], [4], [5]. In early stages of the COVID-19 pandemic, more extensive measures were taken, such as school closures, lockdowns and social distancing, as well as restrictions on public life, to prevent the transmission of the virus [6].

The Standing Commission on Vaccination (STIKO) in Germany provides evidence-based expert recommendations on vaccination against SARS-CoV-2. These recommendations are tailored based on previous vaccinations or infections, age and previous illnesses [7]. The Robert Koch Institute, the German government’s central agency for disease surveillance and prevention, provided recommendations for personal hygiene to protect oneself and others from SARS-CoV-2 infection. Recommendations included keeping distance, washing hands regularly, and wearing a mask [3]. Using the first letters of the German words for these three hygiene measures, this recommendation was disseminated throughout Germany in various campaigns under the acronym AHA [4]. Individuals who knew they were infected with SARS-CoV-2 were asked to notify all contacts they had been in touch with during and 2–3 days before symptom onset, if possible, to facilitate testing and self-isolation if positive [6].

Studies show that adherence to expert vaccination recommendations for SARS-CoV-2, personal hygiene measures [8], [9], and informing contacts of infection [10] can effectively contain and slow the spread of the virus. In contrast to restrictions on public life, such as facility closures, retail stores, cultural venues, and restaurants, the responsibility for implementing these measures in Germany has largely fallen on citizens during this phase of the pandemic. Nonadherence has never been sanctioned by the government. Given the voluntary nature of these measures, understanding factors associated with nonadherence is crucial. This knowledge can inform targeted campaigns to promote the uptake of appropriate measures and their importance in future pandemics.

This study examines potential associations between sociodemographic, health, and virus-related (previous infections, vaccinations) and self-reported nonadherence to recommended individual behaviors for SARS-CoV-2 pandemic containment. Data from the fourth round of the Cologne Corona Surveillance Project (CoCoS), a large cross-sectional survey conducted between December 2022 and January 2023, were utilized for this analysis.

## Methods

### Setting

The survey was conducted in Cologne, Germany, the largest city in North Rhine-Westphalia with a population of 1.08 million, and the fourth largest in Germany [11]. At the time of the survey, 482 thousand PCR-confirmed cases had been reported in Cologne, and approximately 1,150 people had died from or with the virus [12]. The survey was conducted during the sixth wave of COVID-19, which was dominated by Omicron’s BA.5 subline [13]. Countermeasures to protect from infection are specified for the city of Cologne by the government of North Rhine-Westphalia and locally operationalized by a municipal committee [14]. At the time of the survey, masks were mandatory on public transportation and in health care facilities.

### Study design

The study, conducted from 7 December, 2022, to 7 January, 2023, is a cross-sectional study. The survey is the fourth round of the CoCoS project, which is conducted by the University Hospital of Cologne in cooperation with the Health Department of the City of Cologne. Previous rounds set different research priorities and some results have already been published [15], [16], [17], [18]. The implementation of the study has been approved by the Ethics Committee of the University Hospital of Cologne and the North Rhine Medical Association. The study is registered in the German Clinical Trials Register (ID: DRKS00024046).

### Sample

Consenting individuals had to meet the following inclusion criteria to participate in the survey: primary residence in Cologne and age of 18 years or older. No other exclusion criteria applied.

### Study procedures and data collection

A random sample of 30,000 Cologne residents was selected from the municipal registration office using a random generator in the official registration management program (MESO, HSH Soft- und Hardware Vertriebs GmbH, 16356 Ahrensfelde OT Lindenberg). Participants were contacted by post, provided with study details, and given a QR code and online survey link. Participants could complete the survey on a PC, tablet or smartphone. Information on data protection, survey duration, and the option to withdraw consent at any time was provided. Clicking a button provided consent and launched the survey.

### Questionnaire

The questionnaire first collected basic participant information including age, sex, height and weight. Participants rated their income as low, medium or high and if they had a migrant background as well as the number of household members. Further they were asked if they currently had any of the following conditions: hypertension, diabetes, cardiovascular disease, chronic lung disease, an immunodeficiency (yes vs. no), a current or previous cancer diagnosis (yes vs. no). We further inquired previous SARS-CoV-2 infections (yes vs. no) and vaccination status against SARS-CoV-2 (yes vs. no). Participants also reported previous SARS-CoV-2 infections and vaccination status, providing details on infection episodes, diagnostic test used (citizen test, self-test, RT-PCR test), and dates of first and last infection. Regarding SARS-CoV-2 vaccination, they were asked whether they had ever been vaccinated and, if so, whether they had received a basic vaccination or a basic vaccination and one or more booster vaccinations so far. The date of first and last vaccination was asked.

Hygiene adherence to AHA rules (keep distance, wash hands regularly, wear a face mask) was assessed. Additionally, participants indicated their willingness to inform contacts if testing positive for SARS-CoV-2, choosing from options including: “No, nobody”, “Few”, “Some”, “Many”, and “Yes, as far as possible all”.

### Definitions

SARS-CoV-2 infection was confirmed through positive rapid antigen detection test and/or RT-PCR result. Vaccination status included categories of unvaccinated, basic vaccinated, one booster, or multiple boosters. Pre-existing conditions required physician confirmation, with obesity (yes vs. no) defined as a body mass index (BMI) of ≥30 kg/m² according to World Health Organization (WHO) criteria [19], calculated from reported body weight and height. Migrant background referred to the participant or at least one parent not having been born in Germany [20].

The first outcome in the analysis was nonadherence to the STIKO COVID-19 vaccination recommendations, including basic and booster vaccination for individuals aged between 18 and 59, and second booster for those over 60 years of age or at increased risk of severe disease progression. Occupational vaccination recommendations were not collected in this study. The STIKO defined diseases with an increased risk of a severe course of COVID-19, including the conditions surveyed in this study and obesity. Infection with SARS-CoV-2 and vaccination were considered equally immunogenic. Furthermore, if an infection event occurred less than three months before or after vaccination, both events were considered as one immunological event. If the infection event occurred more than three months before or after a vaccination, it was considered a separate immunologic event. Booster vaccinations were supposed to be given more than six months after a previous vaccination or infection unless immunodeficiency is present. In the presence of immunodeficiency, the recommended interval between booster vaccination and the previous vaccination or infection was supposed to be three months [7].

Nonadherence to hygiene recommendations was defined as nonadherence to the so-called ‘AHA rules’ [21] and is therefore widely known accordingly. Nonadherence was considered if a participant answered “no” to any of the three hygiene recommendations.

Adherence to notify all contacts in case of a positive test result was indicated by selecting “Yes, as far as possible all” in the survey. Correspondingly, nonadherence was defined as all other response options to this survey question.

### Statistical analysis

Absolute and relative frequencies of sociodemographic characteristics, health-related, and SARS-CoV-2 specific information were determined in the analyzed datasets. Data on the population of Cologne were obtained from official city statistics and used for comparison to assess cohort representativeness [22], [23].

Outcome variables included nonadherence to STIKO recommendations, nonadherence to hygiene rules (AHA rules), and nonadherence to notifying all contacts after a positive SARS-CoV-2 test. Logistic regression was used to examine associations between each outcome variable and sociodemographic, health, and virus-related variables. The following sociodemographic variables were included in the analysis: age (continuous), sex, self-reported income, personal living situation, and migrant background. The following health and virus-related variables were included into the analysis: obesity, hypertension, diabetes, cardiovascular disease, chronic lung disease, current immunodeficiency, cancer currently being treated or treated in the last year, vaccination status, and previous infections.

Univariable and multivariable logistic regression were performed for each outcome variable. Variables with at least five events in each variable category were included. Prior to multivariable logistic regression, multicollinearity was assessed using the variance inflation factors (VIF) of all variables considered. Variables with VIF>5 were excluded. The multivariable analyses were performed as both a full model and a selected-model, employing stepwise backward selection based on a significance level of p>.10 (Wald statistic). Reported p-values are two-tailed, with p<0.05 considered statistically significant. No adjustments were made for multiple testing (thus accepting a possibly increased false-positive rate) to retain statistical power.

Calculations and figures were performed using SPSS Statistics (IBM Corp., version 29.0.0.0, Armonk, NY, USA), R (R Foundation for Statistical Computing, version 4.3.0, Vienna, Austria), Excel (Microsoft Corp., Redmond, WA, USA), and PowerPoint (Microsoft Corp., Redmond, WA, USA) software.

## Results

### Sample characteristics

**Figure 1:**
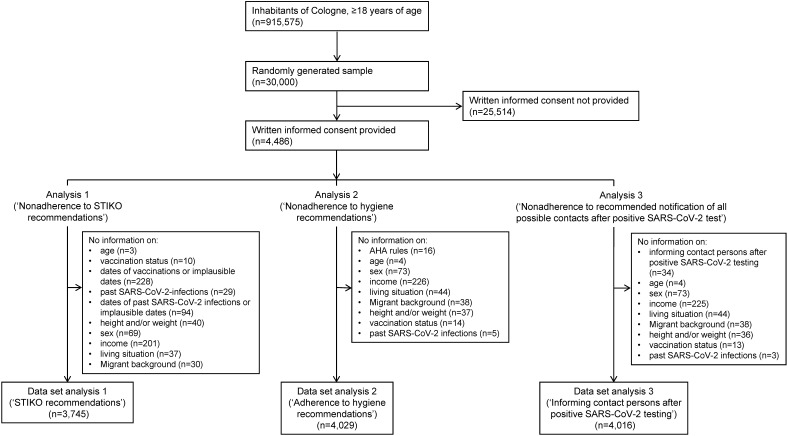
Overview of the recruitment for the fourth round of the CoCoS project, the selection of the cohorts up to the datasets eventually analyzed here

Out of 30,000 invited individuals for the fourth round of the CoCoS project, 4,486 (14.95%) gave written informed consent. Those with missing or implausible data regarding the outcome variable or other analyzed variables were excluded. Consequently, distinct complete case analysis datasets were created for each outcome. The study recruitment flowchart and generation of the three analysis datasets are depicted in Figure 1 (Fig. 1). 

### Description of study participants

Table 1 (Tab. 1) provides a detailed description of the study participants, divided into three analysis data sets and compared with known characteristics of the Cologne population. A statistical comparison between the three data sets revealed no significant differences across analyzed variables (p=.668–.999, see Table 1 (Tab. 1)). Therefore, statements regarding representativeness apply uniformly to all data sets.

The samples are representative in terms of age and sex, but participants with a migrant background were underrepresented compared to the Cologne population. While 37.9% of the Cologne population over 18 years of age has a migrant background, this proportion ranged from 15.6% to 16.0% in the analysis data sets. The vaccination rate in the study sample was significantly higher than in the Cologne population. While 18.5% of the Cologne population had received more than one booster dose, the rate in the study population ranged from 50.1% to 50.4%. In contrast, 9.1% of the Cologne population had not been vaccinated. In the data sets analyzed, the rate was only 1.3% to 1.5%. Direct comparison of infection rates was not feasible because official statistics only record PCR-confirmed cases. No official data were available to compare pre-existing conditions, self-reported income, and personal living conditions. 

### Nonadherence to recommended individual behaviors for SARS-CoV-2 pandemic containment

**Figure 2:**
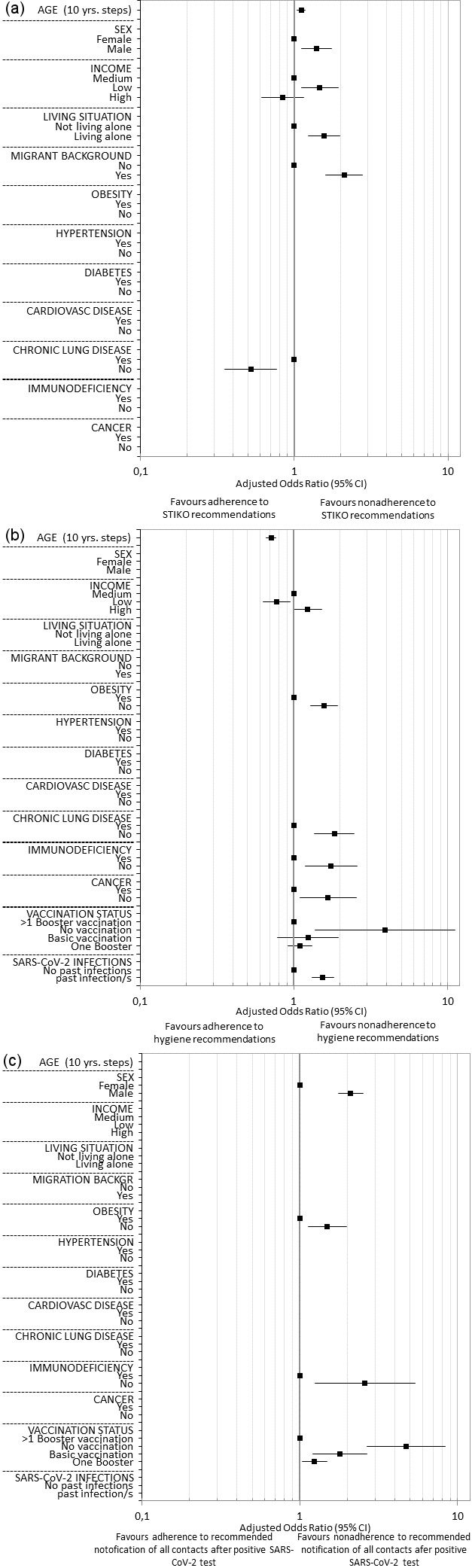
Forest plots of the results of the multivariable (selected model) logistic regressions – (a) associations with nonadherence to STIKO recommendations, (b) associations with nonadherence to hygiene recommendations – (c) associations with nonadherence to recommended notification of all possible contacts after positive SARS-CoV-2 test

**Figure 3:**
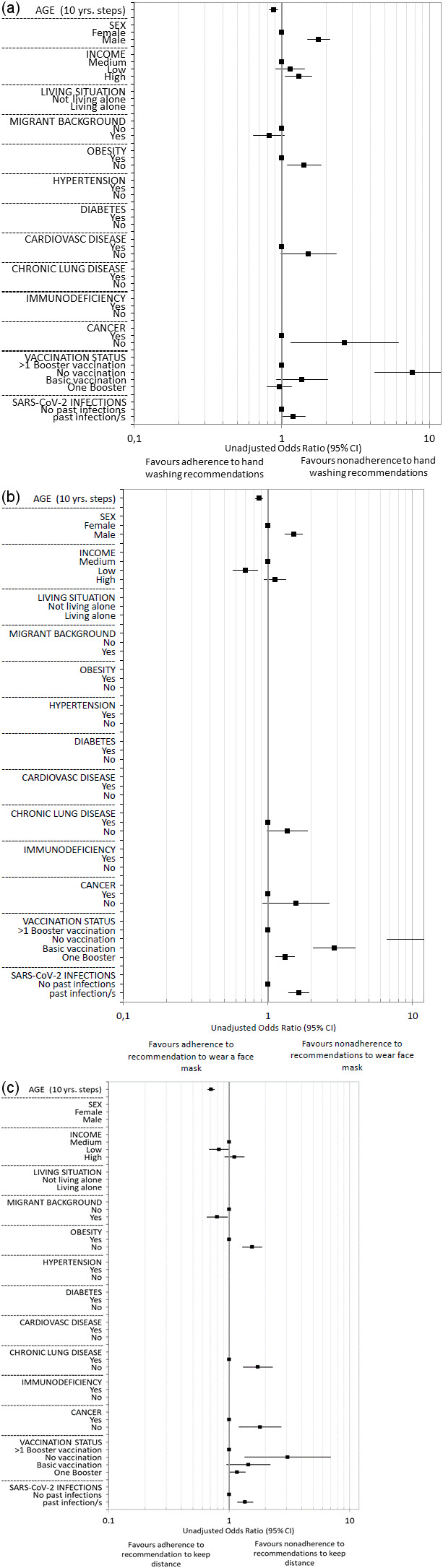
Forest plots of the results of the multivariable (selected model) binary logistic regressions – (a) associations with nonadherence to hand washing recommendations, (b) associations with nonadherence to recommendation to wear a face mask, (c) associations with nonadherence to recommendation to keep distance

The results on the associations between the sociodemographic, health, and virus-related variables surveyed and nonadherence to each behavioral recommendation to contain the SARS-CoV-2 pandemic are presented below. Multivariable logistic regression analyses with variable selection (selected model) are described in the following text and corresponding forest plots are shown in Figure 2 (Fig. 2) and Figure 3 (Fig. 3). Results of the univariable and multivariable analyses without variable selection (‘complete model’) can be found in Table 2 (Tab. 2), Table 3 (Tab. 3) and Table 4 (Tab. 4).

### Nonadherence to STIKO COVID-19 vaccination recommendations

Data from 3,745 participants were analyzed, with all predictors having a VIF value of less than 1.3 as shown in Table 5 (Tab. 5). Out of 4,082 participants, 383 (9.4%) reported nonadherence to STIKO COVID-19 vaccination recommendations.

Regarding sociodemographic variables, age, sex, self-reported income, living situation, and migrant background were significantly associated with nonadherence to the STIKO recommendations. Each year of age increased the odds of nonadherence by 1.1% (OR: 1.011, 95% CI: 1.004–1.018), while men had higher odds than women (adjusted odds: 0.106 vs. 0.076, OR: 1.394, 95% CI: 1.109–1.754). Those with low income were more likely not to adhere to the STIKO recommendation compared to those with medium income (adjusted odds: 0.125 vs. 0.085, OR: 1.464, 95% CI: 1.112–1.928, p=.007). Participants living alone were also more likely not to follow recommendations (adjusted odds: 0.123 vs. 0.079, OR: 1.563, 95% CI: 1.228–1.989), as were those with a migrant background, who had more than double the odds of nonadherence (adjusted odds: 0.166 vs. 0.079, OR: 2.108, 95% CI: 1.608–2.763).

Among health variables, only chronic lung disease was found to be statistically significantly associated with nonadherence to the STIKO recommendations. Participants with chronic lung disease were about twice as likely as participants without chronic lung disease not to comply with the STIKO recommendations (adjusted odds: 0.163 vs. 0.085, OR: 0.522, 95% CI: 0.354–0.770).

No significant associations were found among virus-related variables. All variables not described here were selected from the multivariable model based on a significance level of p>.10 (Wald statistic). 

### Nonadherence to hygiene recommendations (AHA rules)

Data from 4,029 participants were analyzed, with all predictors having a VIF of less than 1.45 (see Table 6 (Tab. 6)). Out of these participants, 3,148 (78.1%) reported not adhering to the hygiene recommendations known as the AHA rules. The breakdown of the hygiene measures into their three sub-components (see Table 7 (Tab. 7), Table 8 (Tab. 8), Table 9 (Tab. 9)) revealed that not maintaining distance from others was the primary reason for the high nonadherence rate to hygiene recommendations. Out of 4,029 participants, 2,903 (72.1%) reported nonadherence to this aspect. Regarding handwashing, only 677 participants (16.8%) reported not usually following this recommendation. As for wearing face masks, 1,255 participants (31.2%) reported not usually following this recommendation.

In terms of sociodemographic factors, both age and self-reported income showed statistically significant associations with nonadherence to hygiene recommendations. With each additional year of age, there was a 2.3% decrease in the odds of nonadherence (OR: 0.971, 95% CI: 0.966–0.976, p<.001). Participants with self-reported low income were significantly less likely to report nonadherence compared to those with medium income (adjusted odds: 3.182 vs. 4.128, OR: 0.771, 95% CI: 0.628–0.946, p=.013). However, the difference between participants with self-reported high income and medium income did not reach statistical significance (adjusted odds: 5.068 vs. 4.128, OR: 1.228, 95% CI: 0.991–1.521, p=.061).

Among health variables, obesity, chronic lung disease, immunodeficiency, and cancer were each statistically significantly associated with nonadherence to hygiene recommendations. Participants with obesity were less likely to report nonadherence to the hygiene recommendations than those without (adjusted odds: 2.789 vs. 4.370, OR: 1.568, 95% CI: 1.279–1.921). Similarly, participants with chronic lung disease (adjusted odds: 2.314 vs. 4.240, OR: 1.833, 95% CI: 1.361–2.467), immunodeficiency (adjusted odds: 2.388 vs. 4.168, OR: 1.745, 95% CI: 1.180–2.581) and cancer (adjusted odds: 2.484 vs. 4.150, OR: 1.671, 95% CI: 1.098–2.543) were less likely to report nonadherence compared to those without these conditions.

In virus-related variables, vaccination status and history of infection were each statistically significantly associated with nonadherence to hygiene recommendations. Unvaccinated participants were significantly more likely to report nonadherence compared to those with multiple booster vaccinations (adjusted odds: 14.965 vs. 3.829), with the odds ratio reaching statistical significance (OR: 3.908, 95% CI: 1.372–11.130, p=.011). However, participants with baseline vaccination but no booster, as well as those with one booster, were only slightly more likely not to adhere to recommendations compared to the reference group with multiple boosters, and these differences did not reach statistical significance (adjusted odds: 4.733 vs. 3.829, OR: 1.236, 95% CI: 0.782–1.954, p=.365/adjusted odds: 4.194 vs. 3.829, OR: 1.095, 95% CI: 0.0918–1.307, p=.312). Participants with no prior SARS-CoV-2 infection were statistically significantly less likely to report nonadherence to hygiene recommendations than participants who had been infected with SARS-CoV-2 virus at least once (adjusted odds: 3.106 vs. 4.779, OR: 1.539, 95% CI: 1.306–1.813).

All other variables were removed from the multivariable model due to a significance level of p>.10 (Wald statistic) as part of variable selection.

### Nonadherence to recommended notification of all possible contacts after positive SARS-CoV-2 test

Data from 4,016 participants were analyzed, with all predictors having VIF values below 1.5 (see Table 10 (Tab. 10) for more details). Among these participants, 625 (15.6%) stated they would not inform all contacts after testing positive for SARS-CoV-2, contrary to recommendations. 

Among the sociodemographic variables, sex was statistically significantly associated with not informing all contacts after a positive SARS-CoV-2 test. Men were twice as likely as women not to follow this recommendation (adjusted odds: 0.255 vs. 0.121, OR: 2.105, 95% CI: 1.765–2.510, p<.001).

Among the health-related variables, obesity and current immunodeficiency were significantly associated. Participants without obesity had 1.5 times higher odds of not informing all contacts after testing positive for SARS-CoV-2 compared to participants with obesity (adjusted odds: 0.181 vs. 0.121, OR: 1.495, 95% CI: 1.133–1.972). Similarly, participants without current immunodeficiency had over twice the odds of not adhering to the recommendation to inform all contacts compared to those with current immunodeficiency (adjusted odds: 0.176 vs. 0.068, OR: 2.588, 95% CI: 1.247–5.369, p=.011).

Among virus-related variables, participants’ vaccination status was statistically significantly associated with nonadherence. Unvaccinated participants had almost five times the odds of not informing all contacts after testing positive, compared to those who had received more than one booster dose (reference) (adjusted odds: 0.700 vs. 0.148, OR: 4.726, 95% CI: 2.669–8.368, p<.001). Participants with completed basic immunization against SARS-CoV-2 had a 1.8-fold increased odds of not informing all contacts compared to the reference (adjusted odds: 0.266 vs. 0.148, OR: 1.793, 95% CI: 1.202–2.676, p=.004), and participants with one booster dose had a 1.2-fold increased odds of not following the recommendation (adjusted odds: 0.184 vs. 0.700, OR: 1.241, 95% CI: 1.035–1.488, p=.020).

All other variables originally included in the model were removed from the model by variable selection based on a significance value of p>.10 (Wald statistic).

## Discussion

This study explores associations between various factors and nonadherence to COVID-19 prevention measures. Results for each recommendation are discussed separately, followed by an analysis of possible patterns. Finally, study strengths and weaknesses are highlighted.

### Nonadherence to STIKO recommendations

The 10% nonadherence rate to STIKO recommendations among respondents does not appear to be a cause for concern. However, the actual nonadherence rate in the general population could be significantly higher, as the sample surveyed is generally more inclined to be vaccinated. The reasons for this are described in the limitations of the study. The STIKO recommendations are grounded in scientific evidence to optimize vaccine effectiveness and minimize risks [7]. Therefore, nonadherence implies suboptimal vaccine protection.

The proportion of participants not adhering to vaccination recommendations during the pandemic suggests a need for intensified efforts in disseminating such guidance in future similar scenarios. This is reinforced by the association between older age and chronic lung disease with higher nonadherence rates. Older age and chronic lung disease correlating with higher nonadherence rates to vaccination recommendations might reflect a lack of awareness regarding the recommendation for a second booster dose in these groups. During the study period, STIKO advised a second booster for individuals over 60 and those with certain pre-existing conditions. Nonetheless, it is unclear why solely chronic lung disease, among other conditions, correlated with higher noncompliance rates to these recommendations. This is all the more surprising as chronic lung disease is known to be a risk factor for severe COVID-19 disease [24], [25], [26] and affected individuals should therefore have a special interest in being up to date with current vaccination recommendations. A possible explanation for this finding may be found in a limitation of the present study. The study did not differentiate between the type and severity of chronic lung disease. For example, otherwise young and healthy participants with mild exercise-induced asthma may have reported having chronic lung disease. However, these participants may not have felt addressed by the STIKO recommendations for a second booster vaccination.

The finding that men are significantly more likely not to follow the STIKO vaccination recommendations reflects the results of studies showing that men are generally less likely to take advantage of disease prevention opportunities [27], [28], [29] and more likely to engage in risky behaviors related to SARS-CoV-2 [30]. More work is needed to understand what can motivate men to change this type of negligent behavior. The finding that individuals living alone in the sample were less likely to follow vaccination recommendations may be related to the public vaccination education strategy. This focused primarily on protecting others [6], [31]. People living alone may be less attracted to such messages and therefore more likely to deviate from vaccination recommendations [32], [33].

The correlation between self-reported low income and increased nonadherence to STIKO recommendations is interesting, given Germany’s free vaccination coverage. Education level might mediate the income-nonadherence link, as lower-income individuals typically have a lower level of education compared to higher-income counterparts [34], [35], [36]. Although the STIKO recommendations are freely available, they are written in technical language and the vaccination algorithm itself is not easy to understand [7]. Future studies should investigate this to avoid exacerbating the already significant health disadvantages of socially disadvantaged groups. Based on observation the city of Cologne deployed mobile vaccination units to areas of the city where people with lower income live.

In Germany, COVID-19 vaccination recommendations were shared in the primary languages of the largest migrant communities. An initiative by the German government in early 2022 promoted SARS-CoV-2 vaccination in Arabic, English, Russian, and Turkish. This approach was intended to reach people with insufficient knowledge of German, including refugees [37]. Language barriers alone may not fully explain the link between migrant background and nonadherence to STIKO vaccination recommendations. Numerous studies indicate that individuals with a migrant background often exhibit lower confidence in vaccination overall, including against SARS-CoV-2, which may explain the observed results [38], [39], [40]. Other studies have shown that people with a migrant background make less use of a wide range of preventive measures and screening tests and have lower vaccination rates than people without a migrant background [41], [42]. Intensive research is needed to determine which factors mediate this phenomenon and how these factors can be changed so that people with a migrant background make use of the services offered by the health care system.

The COVIMO study examines vaccination readiness and acceptance in Germany [43]. It shows that concerns about possible side effects and doubts about the effectiveness of vaccines are potential reasons for reluctance to be vaccinated. In addition, people who feel urged to be vaccinated tend to be less willing to be vaccinated. This may have contributed to the reluctance of the unvaccinated, especially during periods when restrictions were placed on the participation of the unvaccinated. Attitudes to vaccination have changed during the pandemic. The COSMO study shows that confidence in vaccination has decreased over time [44]. Compared to April 2020, more people think vaccination is unnecessary. In addition, more people are weighing the benefits against the potential risks and feel that vaccination is unnecessary if other people have already been vaccinated.

### Nonadherence with hygiene recommendations (AHA rules)

Paragraph 2 of the Corona Protection Order appealed to the public to follow the AHA rules responsibly and in solidarity to avoid putting themselves and others at risk of SARS-CoV-2 infection [45]. Obviously, people find it particularly difficult to follow the recommendation to keep their distance from other people. The high percentage, approximately 70%, of participants not adhering to this recommendation contributes to the overall figure of nearly 80% of participants not following the hygiene “AHA” recommendations. This underscores a key challenge for future pandemic responses: finding ways to convey the importance of temporary isolation and distancing while addressing individuals’ essential need for human interaction and connection.

Our research found that certain health conditions, including obesity, chronic lung disease, current immunodeficiency, and cancer, were significantly associated with nonadherence to the recommended AHA rules. Notably, only presumed serious pre-existing conditions of chronic lung disease, current immunodeficiency, and cancer showed statistically significant associations with nonadherence to hygiene recommendations, while presumed less serious conditions like hypertension, cardiovascular disease, and diabetes did not. Research indicates that early stages of cardiovascular disease and diabetes, along with obesity, are linked to higher risks of severe disease progression [46], [47]. This finding could be interpreted as a cue to increase public education about these issues.

The finding that higher-income participants were more likely not to follow the AHA rules than middle- or low-income participants raises questions. Adherence to hygiene recommendations is an effective strategy for limiting the spread of pathogens such as SARS-CoV-2 [8], [9]. That higher-income individuals in our sample appear to be less inclined to follow these simple measures is disconcerting and requires further confirmation. Future studies should further elucidate the underlying motives and mediators in different social classes.

The significant association found here between prior SARS-CoV-2 infection and non-adherence to the AHA rules warrants discussion. It is possible that those who have had the virus believe they are immune and thus neglect hygiene measures, a misconception that overcoming an infection permanently protects against reinfection [48]. Future research should explore this and focus on correcting such misperceptions in risk communication.

### Nonadherence to recommended notification of all possible contacts after positive SARS-CoV-2 test

It is advised to monitor for SARS-CoV-2 symptoms post-contact to prevent transmission [5]. Therefore, informing all contacts of a positive test result is crucial. The 15% nonadherence rate among participants is concerning.

In this study, men were twice as likely as women not to inform all contacts if possible after a positive SARS-CoV-2 test result. Similar to testing for flu-like symptoms, this may be due to lower health awareness among men compared to women [49]. Those with multiple booster shots were significantly more likely to inform all contacts of a positive SARS-CoV-2 test compared to those with fewer shots, indicating better adherence to infection control measures. Studies have shown that persons with multiple vaccinations have better health awareness than persons with fewer vaccinations [50]. In addition, vaccinated individuals may be better informed about the risks of infection and therefore more motivated to follow recommended behaviors.

### Patterns of tendency not to follow recommendations

Men were significantly more likely than women not to comply with individual behavioral recommendations to mitigate the SARS-CoV-2 pandemic in two of three analyses. Only for personal hygiene did men not show a greater tendency to nonadherence compared with women. 

One out of three analyses showed a significant tendency for participants who reported low income not to follow recommendations for SARS-CoV-2 pandemic containment. This related to STIKO vaccination recommendations.

Pre-existing conditions generally appeared to be associated with adherence to expert recommendations, but no clear pattern emerged for specific pre-existing conditions. The only exception was the tendency of those with chronic lung disease not to follow STIKO vaccination recommendations. 

In all analyses presented here, lack of vaccination against COVID-19 was significantly associated with nonadherence to expert recommendations.

### Strengths and limitations of the study

A strength of the present study is the large number of randomly selected participants (n=4,486). In addition, the present study was able to obtain detailed information on nonadherence to recommended individual behaviors to mitigate the SARS-CoV-2 pandemic. For the first time, these were related to socio-demographic, health and virus-related characteristics.

One limitation of the study is potential selection bias, particularly in certain age groups. Participants under 34 and over 75 years old were underrepresented, while those between 35 and 74 were overrepresented compared to the general adult population of Cologne. This may stem from younger individuals being less responsive to mail invitations and older individuals being less likely to participate in online surveys. Older people are often less comfortable with the Internet than younger people [51]. To enhance representativeness across age groups in future studies, alternative communication methods could be explored. Recruiting younger participants via email lists of university networks and social media advertising could be effective. Additionally, mailing paper questionnaires and conducting surveys in assisted living and nursing homes should be considered.

A potential selection bias existed concerning migrant background. Those with a migrant background were notably underrepresented compared to Cologne’s general adult population. Offering the questionnaire in English and Turkish did not notably boost participation among this group in previous rounds of the CoCoS project [18]. Promoting participation in studies like this one and emphasizing their significance for effective pandemic control might enhance acceptance and increase participation rates among those with a migrant background.

Potential selection bias also occurred with respect to the proportion of people with SARS-CoV-2 vaccination or booster(s). In the present study, the proportion of unvaccinated persons was strongly underrepresented compared to the general adult population of Cologne. Refusal to vaccinate is highly correlated with refusal to take measures to contain the spread of SARS-CoV-2 and with a general attitude of hostility towards the issue [52], [53]. This would also imply that the estimates of nonadherence to individual behavioral interventions reported here underestimate the true magnitude. The voluntary nature of the survey and absence of incentives likely attracted individuals more interested in COVID-19 topics and measures. Offering financial or other incentives could encourage participation from individuals with less interest or declining attitudes in future studies. For example, the Robert Koch Institute uses raffles among participants as an incentive to make participation in platforms such as Flu Web more attractive [54].

Considering the low participation rate in our study, there is a possibility of self-selection, which could affect representativeness. Although the sample is not representative, it nevertheless provides important insights that can contribute to the discussion about how to make people adhere to personal behavioral measures in reducing the spread of the virus in a pandemic situation.

Future studies should address the problem of lack of representativeness using statistical weighting methods. Such methods are commonly used by commercial survey agencies. However, such methods require even more detailed information on socio-demographic and socio-economic characteristics [55], [56].

A possible recall bias is debatable, especially with regard to the data on infections and vaccinations used to determine nonadherence to the STIKO vaccination recommendations. However, it can be assumed that infections and vaccinations are remembered as formative events, so that at least the month and year of these events can be recalled. In addition, this information is easily accessible through apps, which have been widely used to check vaccination status [57].

In addition, social desirability may mean that actual nonadherence is even higher than reported. In addition, the survey was conducted at a late stage of the pandemic, which could be another explanation for increased nonadherence. This issue has already been discussed in the context of the STIKO vaccination recommendations [44] and could apply in a similar way to adherence to hygiene recommendations and contact tracing.

Another limitation is the categorization of behaviors, which focused on strict nonadherence versus adherence. Treating nonadherence as binary limits understanding. Future studies should explore nonadherence as a spectrum. Nevertheless, our binary analysis offers trend indications supporting our statements.

Lastly, the study’s limitation lies in its localized scope within an urban setting in Germany. Expanding the catchment area could enhance the validity of similar studies.

## Conclusion

The study offers key insights into how sociodemographic, socioeconomic, health, and virus-related factors relate to nonadherence with SARS-CoV-2 containment recommendations. Findings indicate that males, lower-income individuals, vaccine refusers, and those with prior SARS-CoV-2 infection were less likely to follow expert guidelines for pandemic control. These results can inform targeted educational campaigns and public communication efforts in future pandemics. Further research should delve into the underlying reasons for these associations, aiding in the optimization of education and public messaging strategies.

## Abbreviations

- CoCoS study: Cologne Corona Surveillance Study
- SARS-CoV-2: Severe acute respiratory syndrome coronavirus 2
- COVID-19: Coronavirus Disease 19
- DRKS: German Clinical Trials Register
- STIKO: Standing Committee on Vaccination
- RT-PCR: Reverse transcription-polymerase chain reaction
- AHA: Keep your distance, wash your hands regularly, wear a face mask
- VIF: Variance inflation factor
- WHO: World Health Organization
- OR: Odds ratio
- CI: Confidence interval

## Notes

### Ethics approval and consent to participate

This study was approved both by the Ethics Committee of the Medical Faculty of the University of Cologne (reference number 20-1685_4) and by the Ethics Committee of the North Rhine Medical Association (reference number 2020447). All methods were carried out in accordance with relevant guidelines and regulations. The trial was registered at https://www.drks.de (ID: DRKS00024046) prior to enrolment. Participants were informed about the risks and potential benefits of the study. Participation was voluntary. Written informed consent was obtained from all participants.

### Availability of data and material

The datasets used and/or analysed within the current study are available from the corresponding author upon reasonable request.

### Funding

German Federal Ministry of Education and Research (“Nationales Netzwerk Universitätsmedizin” grant number 01KX2021). The funding body did not play a role in the design of the study and collection, analysis, nor in interpretation of data or in writing the manuscript.

### Authors’ contributions

MO and KB have written the manuscript. MH, FN, AK, JR, TA, FD, MB and KS contributed to the manuscript development and provided scientific expertise. MH, KS, TA, FN, KR, KB and MO designed the statistical procedures. All authors read and approved the final manuscript.

### First and last authorship

MO and KB share first authorship. FN and MH share last authorship.

### Acknowledgements

The authors would like to thank all participants in the study for filling out our questionnaire. We thank the City of Cologne (Stadt Köln) for support with drawing the random sample of Cologne residents.

### Competing interests

The authors declare that they have no competing interests.

## Figures and Tables

**Table 1:**
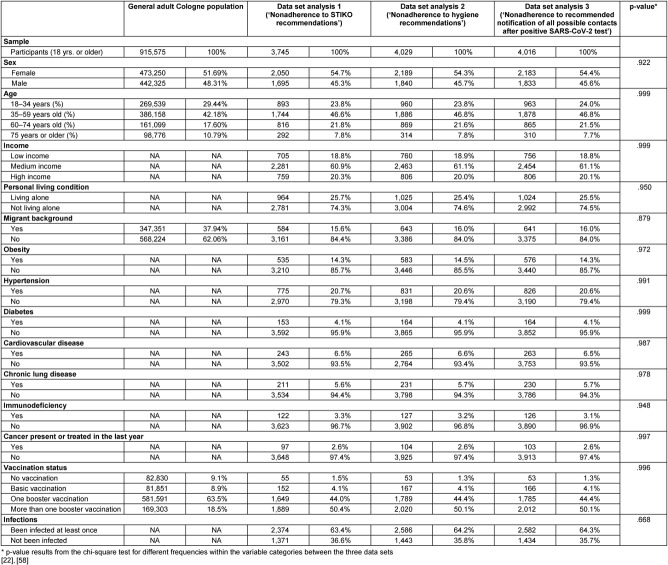
Sociodemographic characteristics, health-related and SARS-CoV-2 specific information of the analyzed cohorts compared to the general adult Cologne population

**Table 2:**
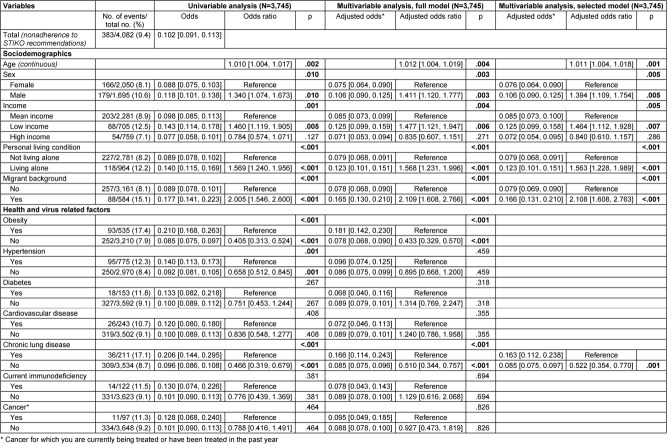
Results of univariable and multivariable (full and selected model) binary logistic regression on nonadherence to STIKO recommendations

**Table 3:**
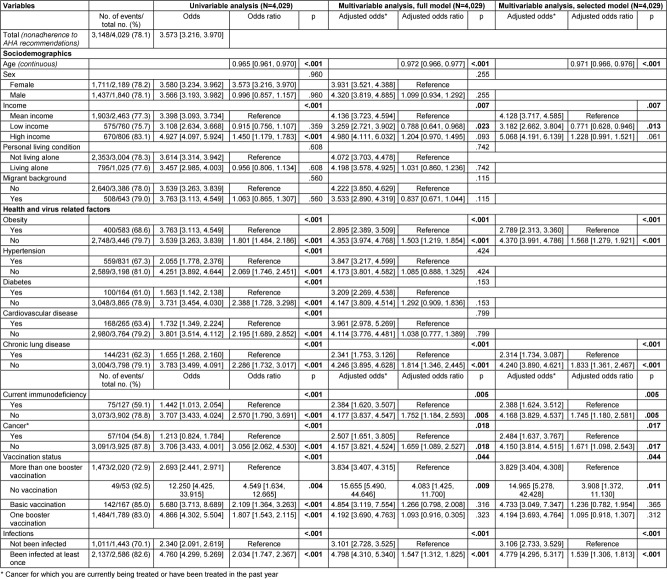
Results of univariable and multivariable (full and selected model) binary logistic regression on nonadherence to hygiene recommendations (AHA recommendations)

**Table 4:**
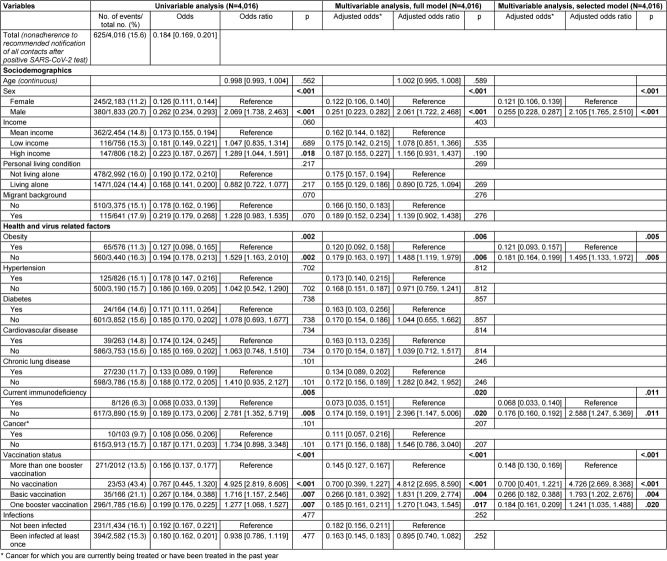
Results of univariable and multivariable (full and selected model) binary logistic regression on nonadherence to recommended notification of all contacts after positive SARS-CoV-2 test

**Table 5:**
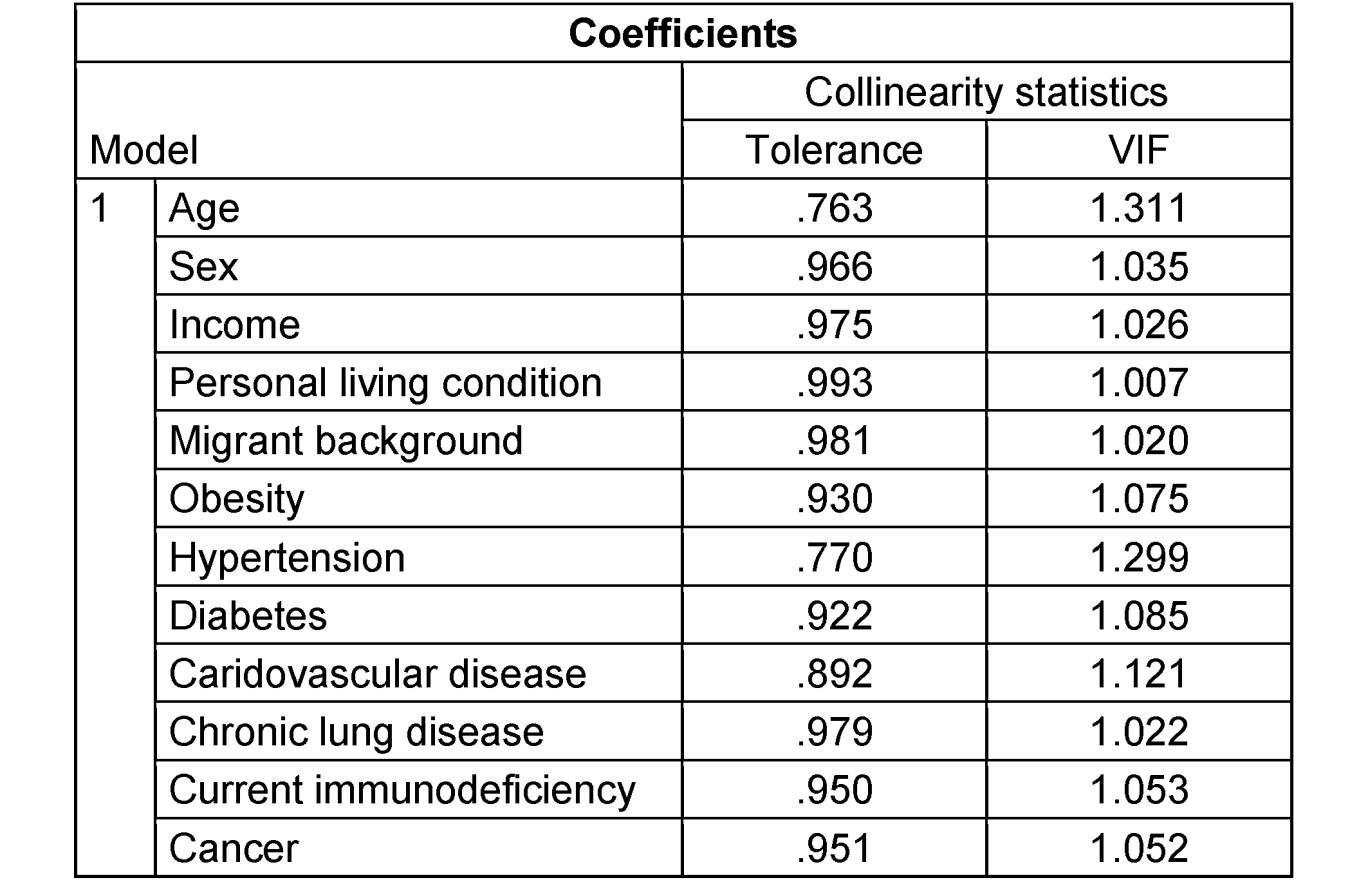
Multicollinearity assessment concerning multivariable analysis of associations with nonadherence to STIKO recommendations using tolerance/variance inflation factor (VIF)

**Table 6:**
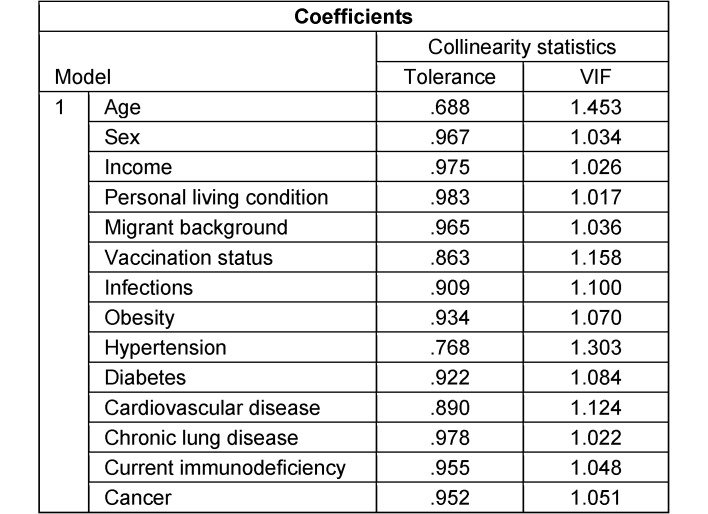
Multicollinearity assessment concerning multivariable analysis of associations on nonadherence to hygiene recommendations (AHA recommendations) using tolerance/variance inflation factor (VIF)

**Table 7:**
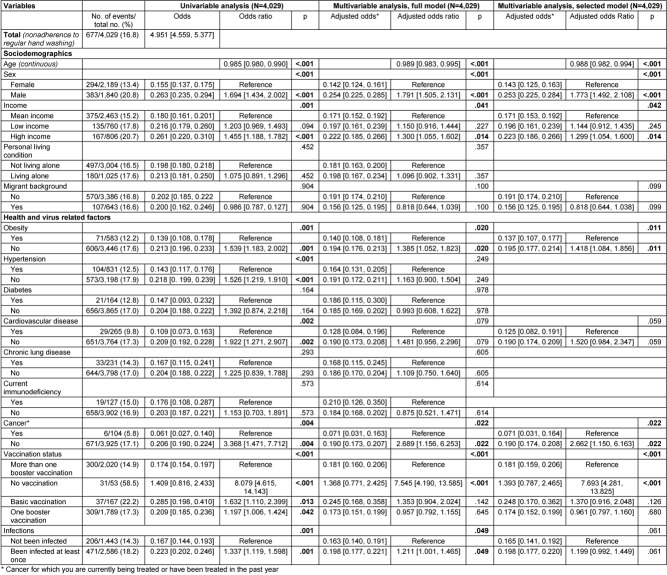
Results of univariable and multivariable (full and selected model) binary logistic regression on nonadherence to recommended regular hand washing

**Table 8:**
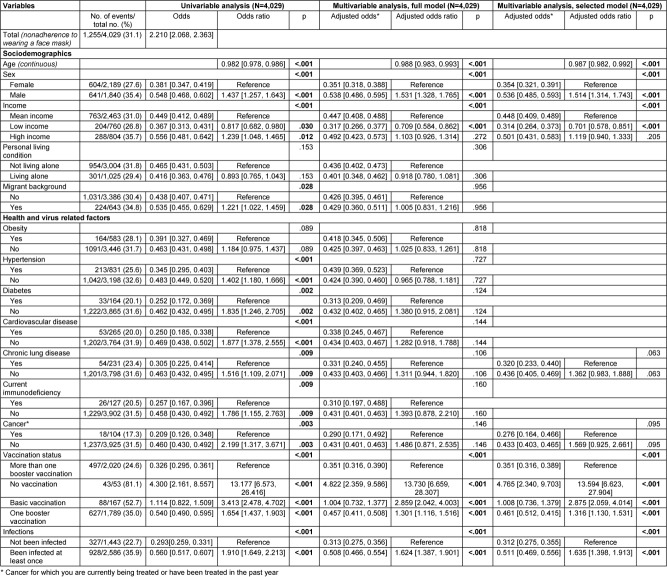
Results of univariable and multivariable (full and selected model) binary logistic regression on nonadherence to wearing a face mask

**Table 9:**
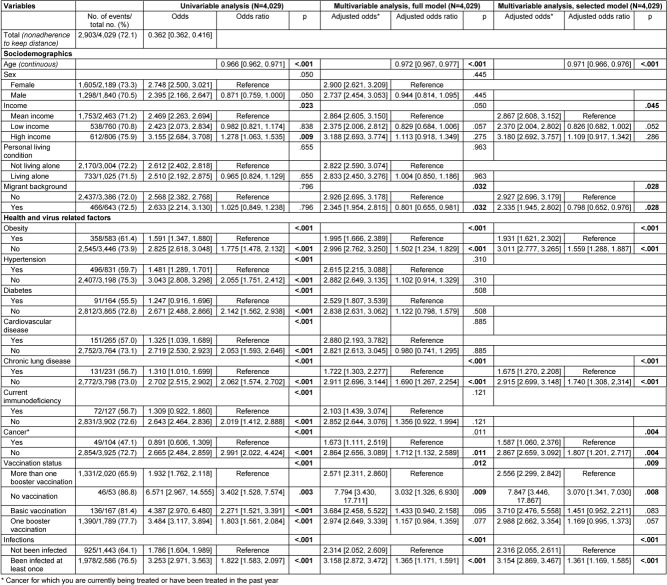
Results of univariable and multivariable (full and selected) binary logistic regression on nonadherence to keeping distance

**Table 10:**
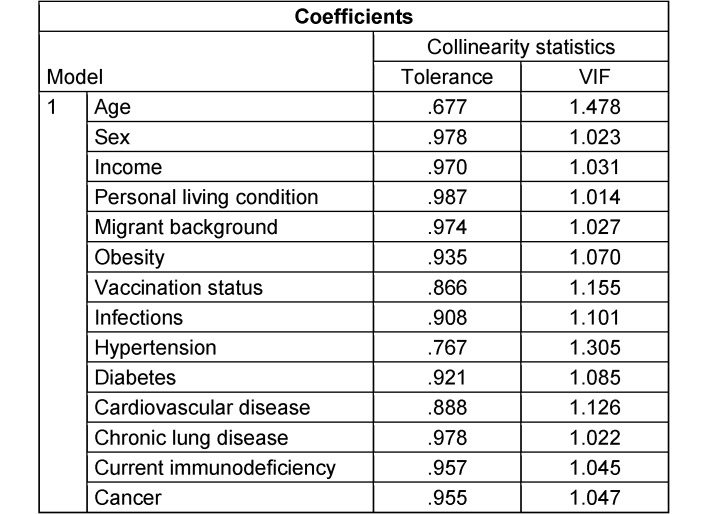
Multicollinearity assessment concerning multivariable analysis of associations on nonadherence to recommended notification of all contacts after positive SARS-CoV-2 test using tolerance/variance inflation factor (VIF)
